# Design of a Wearable Exoskeleton Piano Practice Aid Based on Multi-Domain Mapping and Top-Down Process Model

**DOI:** 10.3390/biomimetics10010015

**Published:** 2024-12-31

**Authors:** Qiujian Xu, Meihui Li, Guoqiang Chen, Xiubo Ren, Dan Yang, Junrui Li, Xinran Yuan, Siqi Liu, Miaomiao Yang, Mufan Chen, Bo Wang, Peng Zhang, Huiguo Ma

**Affiliations:** 1 School of Arts and Design, Yanshan University, Haigang District, Qinhuangdao 066000, China; xuqiujian@ysu.edu.cn (Q.X.); cgq9691@ysu.edu.cn (G.C.); lijunrui_1023@163.com (J.L.); yuanxinran0112@163.com (X.Y.); liusiqi19990919@163.com (S.L.); yangmiaomiao07@163.com (M.Y.); chenmufan0921@163.com (M.C.); 2 YSU & DCU Joint Research Centre for the Arts, Music College, Daegu Catholic University, Daegu 38430, Republic of Korea; 18686969513@163.com (M.L.); xiuboren@gmail.com (X.R.); yangdan6413@163.com (D.Y.); 3 Hebei Design Innovation & Industrial Development Research Center (DIIDRC), Yanshan University, Haigang District, Qinhuangdao 066000, China; kstg4568320@gmail.com; 4 Department of Design, Kyungpook National University, Daegu 41566, Republic of Korea; 5 School of Information Engineering, Quanzhou Ocean Institute, Quanzhou 362700, China; zhwdrfcdaaerov@hotmail.com

**Keywords:** multi-domain mapping, layout solution, wearable exoskeleton, piano practice assistance device

## Abstract

This study designs and develops a wearable exoskeleton piano assistance system for individuals recovering from neurological injuries, aiming to help users regain the ability to perform complex tasks such as playing the piano. While soft robotic exoskeletons have proven effective in rehabilitation therapy and daily activity assistance, challenges remain in performing highly dexterous tasks due to structural complexity and insufficient motion accuracy. To address these issues, we developed a modular division method based on multi-domain mapping and a top-down process model. This method integrates the functional domain, structural domain, and user needs domain, and explores the principles and methods for creating functional construction modules, overcoming the limitations of traditional top-down approaches in design flexibility. By closely combining layout constraints with the design model, this method significantly improves the accuracy and efficiency of module configuration, offering a new path for the development of piano practice assistance devices. The results demonstrate that this device innovatively combines piano practice with rehabilitation training and through the introduction of ontological modeling methods, resolves the challenges of multidimensional needs mapping. Based on five user requirements (P), we calculated the corresponding demand weight (K), making the design more aligned with user needs. The device excels in enhancing motion accuracy, interactivity, and comfort, filling the gap in traditional piano assistance devices in terms of multi-functionality and high adaptability, and offering new ideas for the design and promotion of intelligent assistive devices. Simulation analysis, combined with the motion trajectory of the finger’s proximal joint, calculates that 60° is the maximum bending angle for the aforementioned joint. Physical validation confirms the device’s superior performance in terms of reliability and high-precision motion reproduction, meeting the requirements for piano-assisted training. Through multi-domain mapping, the top-down process model, and modular design, this research effectively breaks through the design flexibility and functional adaptability bottleneck of traditional piano assistance devices while integrating neurological rehabilitation with music education, opening up a new application path for intelligent assistive devices in the fields of rehabilitation medicine and arts education, and providing a solution for cross-disciplinary technology fusion and innovative development.

## 1. Introduction

### 1.1. Current Status of Piano Learning

Piano learning is not only a form of artistic expression but also a process that involves complex muscle coordination and multi-stage learning [1]. For patients with stroke and other neurological disorders, piano learning can serve as a beneficial tool in their rehabilitation process, particularly in promoting neuroplasticity and restoring hand function [2]. However, these patients face unique challenges during the learning process due to weakened limb function and decreased coordination abilities. Although standard piano teaching resources are abundant, including online and offline courses, as well as one-on-one and group instruction, these resources are often not tailored to the specific needs of neurorehabilitation patients [3,4]. Research has shown that appropriate technological interventions can significantly improve the rehabilitation outcomes for these patients [5]. In this context, the development of a wearable exoskeleton piano practice assistance device specifically designed for patients with stroke and other neurological disorders becomes particularly important. This device physically assists patients in correctly positioning their fingers and adjusting force during piano playing. Additionally, it utilizes integrated sensor technology to provide real-time feedback, promoting the formation of correct hand positioning and the establishment of muscle memory. At the same time, incorrect hand positioning and techniques can not only affect the quality of performance but also lead to excessive stress on the fingers and wrists, exacerbating the patient’s pain and discomfort [6]. It is crucial to correct these issues promptly, yet traditional teaching methods often have limited effectiveness in this regard. The assistance of smart exoskeleton devices enables patients to independently practice correctly without the need for prolonged external supervision, effectively reducing the physical strain caused by improper hand positioning [7]. Therefore, the wearable exoskeleton piano practice assistance device offers an innovative training method for neurorehabilitation patients. It not only enhances patients’ confidence and motivation to learn but also significantly improves rehabilitation efficiency by increasing the fun and interactivity of the learning process. The development and application of this device are expected to garner widespread attention and research in the field of neurorehabilitation, providing patients with more effective and humanized rehabilitation support. Additionally, it offers multifaceted benefits for students, parents, teachers, and training institutions, demonstrating its broad social applicability.

### 1.2. Research on the Application of Exoskeleton Technology

Exoskeleton technology has garnered widespread attention due to its significant effectiveness in rehabilitation therapy, particularly in the rehabilitation of elderly patients [8,9]. By simulating limb movements, this technology makes the treatment process more autonomous and intelligent. The extensive application of exoskeleton technology in clinical practice not only demonstrates the robust growth of the rehabilitation assistance industry but also strengthens public recognition and trust in the use of this technology for hand rehabilitation [10,11,12,13].

**Figure 1:**
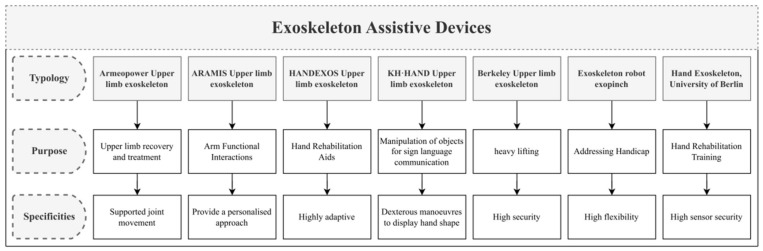
Classification diagram of various exoskeleton assistive devices.

Although exoskeleton technology has achieved some success in the rehabilitation field, its application in education, particularly in piano teaching, is still in its early stages. Traditional piano teaching assistance devices, such as those that use passive electrical stimulation, may cause physiological and psychological discomfort for learners, including physical fatigue during practice, a lack of confidence, and improper hand positioning. To address these issues, the development of new piano teaching assistance devices has emerged, not only filling a gap in the market demand but also effectively promoting hand training and enhancing the learners’ practice experience. These innovative devices are expected to provide more creative and beneficial solutions in the field of piano education, driving the integrated development of technology and education (as shown in Figure 1).

### 1.3. Application of Piano Exoskeleton Assistance Devices

Regarding the current application of wearable exoskeleton piano practice aids, these devices primarily support piano learning and rehabilitation therapy by simulating and enhancing hand movements [14]. In recent years, significant progress has been made in the development of piano assistance technology for assistive devices. For example, the soft robotic glove developed by Florida Atlantic University is designed to help stroke patients relearn piano skills. This glove provides sensory feedback and mechanical assistance, significantly improving motor control and rehabilitation outcomes [15]. Additionally, “The Piano Press Glove”, designed for patients with rheumatoid arthritis, enhances key-pressing ability through a cable-driven system, combining physical therapy with piano playing. This device aims to provide an enjoyable therapeutic experience while alleviating joint pain [16]. Further research has evaluated exoskeleton gloves that impose fewer restrictions on the free movement of pianists’ fingers and enable faster movement. After prolonged passive movement guidance, even when the gloves are removed, the variation in key-pressing force during performance is significantly reduced. This passive movement aids in finely controlling force output [17]. Research on new hand exoskeleton robots has shown that continuously correcting finger movements and reducing the load can improve finger technique. The study collected initial data using finger joint angle sensors to analyze the movements during piano playing, with a particular focus on the trajectory and angular velocity of key strikes [18]. These advancements demonstrate how piano assistance technology has evolved into a complex system integrating sensors and actuators, not only supporting musical performance for individuals with neurological disorders but also playing a critical role in rehabilitation therapy. This evolution fully reflects the deep integration of technology and user needs. Despite this, the significant achievements of piano assistance technology in helping people with disabilities learn and play the piano, there are still some limitations in structural design and human–computer interaction. From a structural perspective, complex designs may reduce the reliability and adaptability of the devices; some equipment may be too bulky, affecting user comfort during prolonged use. In terms of human–computer interaction, although there have been improvements in the intuitiveness and ease of operation, the timeliness and accuracy of feedback still require further optimization. Additionally, the flexibility of personalized settings is insufficient, making it difficult to meet the specific needs of all users. Future developments should focus on addressing these issues to create a higher-quality user experience.

**Figure 2:**
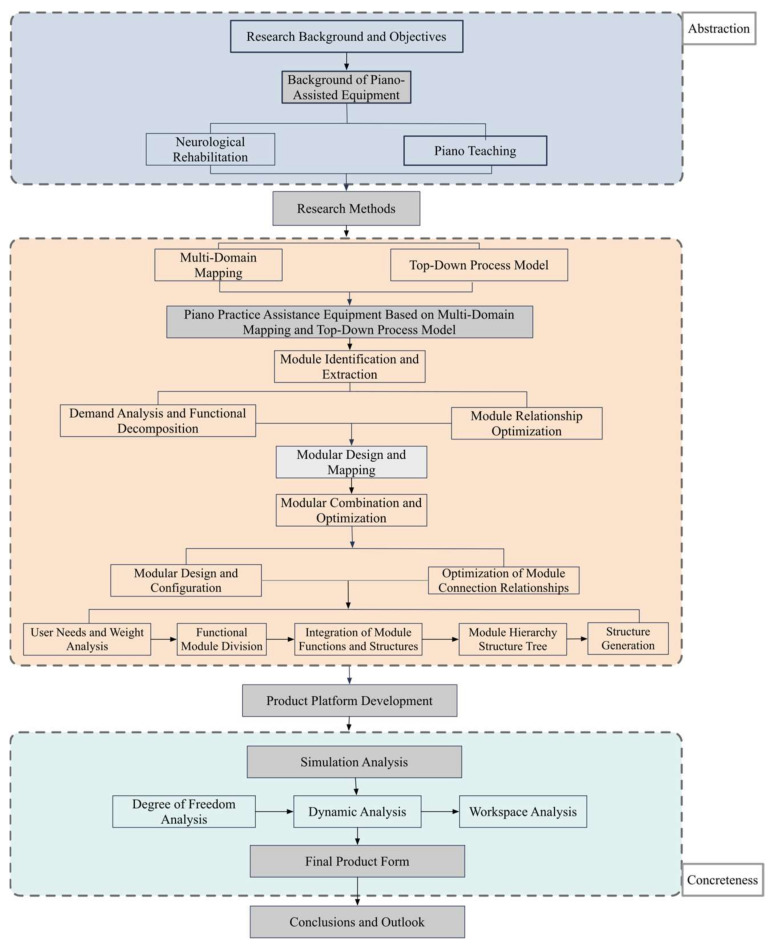
Schematic diagram of the piano practice assistance device design.

To address the aforementioned issues, it is necessary to develop a design method that combines multi-domain mapping with a top-down process model to optimize the design of piano practice assistance devices. The current multi-domain mapping layout units exhibit certain irregularities in constructing hierarchical relationships and topological structures, which affects the effective storage and retrieval of information [19]. To resolve this issue, this study adopts an ontological definition and structural form, systematically constructing relationships between different levels and dimensions through an ontological approach. This method optimizes the hierarchical topological structure of layout units, making it more intuitive and organized, thereby aiding in the construction of a comprehensive database that includes functional, motion, and structural relationships, supporting knowledge accumulation, retrieval, and multi-type relationship storage. Based on this theoretical framework, this study further develops a multi-domain mapping layout configuration solution model that integrates functional requirements, motion allocation, and functional-structural carriers. This model not only derives the mapping of associated hierarchical relationships but also provides critical information support for constructing the hierarchical topological structure of layout units. It effectively realizes the mapping from function to structure and optimizes the solution process of the layout configuration. Additionally, by combining forward design and layout constraints, this study establishes a layout morphology diagram for the assembly skeleton model. Through the analysis of the correlation between the top-down design process and layout constraints, the study establishes the assembly logic of the layout structure modules and emphasizes the importance of constructing the assembly skeleton model. By adopting a specialized tree diagram method, the study further refines the construction process of the assembly skeleton model for the piano practice assistance equipment, providing the necessary spatial constraint information for the automatic assembly of layout structure modules. The visualization of the layout morphology diagram offers a visual reference for optimizing the layout configuration, as shown in Figure 2.

### 1.4. Application of Top-Down Design in Mechanical Design

As is well known, in the long history of mechanical design, top-down product design has always been a critical issue, and the computerized tools that support top-down design are indispensable [20]. In conceptual design, there are many methods and techniques available for establishing functional structures. Sturges and others proposed functional flowcharts and functional logic diagrams to represent functions [21]. Umeda and Karnopp, along with others, introduced the Function-Behavior-State (FBS) model, which links functional symbols, behavior, and state. In this model, functional symbols represent the subjective aspect, while behavior and state represent the objective aspects [22]. In the top-down product design approach, the contributions of various scholars highlight the depth and breadth of this field. For instance, Pahl and Beitz extensively discussed systematic methods in the design process, which aid in progressively refining the product from functional requirements to concrete implementation. Additionally, Hubka and Eder explored advances in design theory, emphasizing the importance of clearly defining the relationships between function, behavior, and state in the early stages of design [23].

Moreover, Suh introduced the theory of axiomatic design, stressing the significance of clearly distinguishing and optimizing design parameters during the decision-making process [24]. Otto and Wood proposed a design method that focuses on defining clear design specifications in the initial stages, with iterative refinement to meet these specifications [25]. Furthermore, Ulrich and Eppinger examined how to effectively implement top-down design strategies in the product development process to ensure the functionality and market adaptability of the design outcomes [26].

This recent research has provided new perspectives for this field. For example, Alexsteven Dharmdas and others proposed the concept of active curvature deformation based on bio-inspired structures, validating the aerodynamic elasticity effects of wing trailing-edge deformation through experimental and simulation methods [27]. This design approach not only emphasizes the importance of lightweight and actively deformable structures but also demonstrates its potential in addressing multi-task requirements, especially in optimizing the implementation of complex functions, showcasing its versatility. Similarly, Arun Y. Pati and others proposed various structural designs based on bio-inspired principles, validating through experiments and additive manufacturing the significant effects of these structures in enhancing impact resistance. These studies have inspired new methods for product component optimization, providing crucial references for the design of complex multifunctional systems [28].

These studies highlight the effectiveness and importance of top-down design methods in practical applications. However, while the top-down product design approach has the advantages of being structured and systematic, it also has certain limitations in real-world applications. First, this approach may overly rely on the initial stage’s requirement definitions and functional planning, neglecting new information and changes that may arise during the design process, which can lead to a lack of flexibility. Second, the top-down method may involve fewer feedback adjustments from underlying details when dealing with complex or unknown problems. Additionally, product design schemes based on functional matching only provide part of the conceptual design’s basic modules, without considering the mapping relationships between modules and the hierarchical structure between concepts, nor do they establish a multi-level hybrid solution framework for the product.

Therefore, it is necessary to establish a wearable exoskeleton piano practice assistance device design method based on multi-domain mapping and top-down processes. This method should research ontology-based product innovation design approaches, product design knowledge models, design knowledge retrieval, and reasoning technologies, and mechanisms for expressing and solving design knowledge. By combining the lightweight deformable structure design proposed by Alexsteven Dharmdas and the bio-inspired structural optimization concept proposed by Arun Y. Pati, we can propose a scientifically sound and rational module configuration scheme for piano practice assistance devices, providing innovative and practical solutions to complex design problems.

## 2. Materials and Methods

### 2.1. Module Identification Process

Conceptual design is a process of gradual refinement, evolving from rough to detailed, from vague to clear, and from abstract to concrete. It centers around the design concept and permeates the entire design process. In today’s rapidly changing market environment, designers face the challenge of transforming innovative ideas into tangible products [29]. This process requires a deep understanding of design principles and the flexibility to adapt the design process to the constantly changing technologies and user needs. Conceptual design, as a critical phase, serves as a bridge that concretizes innovative concepts and ensures a smooth transition from the conceptual stage to the development stage [30]. Modularizing the product design, with its flexible structural configuration, not only optimizes the design process but also enhances the adaptability and market responsiveness of the product. This modular approach plays a central role in modern product design [31].

By combining multi-domain mapping with a top-down process model, the modular design supports designers in effectively integrating information and making decisions across different functional and structural domains. These two approaches form a powerful framework that ensures diversity and flexibility in design solutions while addressing complex market demands. In product design, module identification plays a crucial role in enhancing users’ understanding and perception of product functions. However, existing methods for identifying modules have certain shortcomings, such as confusing expression mechanisms that make the objective description of functions difficult. This challenge arises primarily because functions often rely on the intuitive understanding of designers or users, and the representation of functions may struggle to fully capture the user’s subjective intentions. Similarly, the representation of behavior cannot always be objectively defined according to the functional model. To address these issues, this study emphasizes the importance of modular design thinking. This approach not only encompasses the gradual understanding of product structure but also accompanies every stage of the product’s lifecycle, from design to deployment. The modular design achieves diversity and flexibility in product design solutions by flexibly combining various modules. The modular design process includes two fundamental steps: module identification and module configuration, with module identification serving as the foundation and prerequisite for the entire design process.

**Figure 3:**
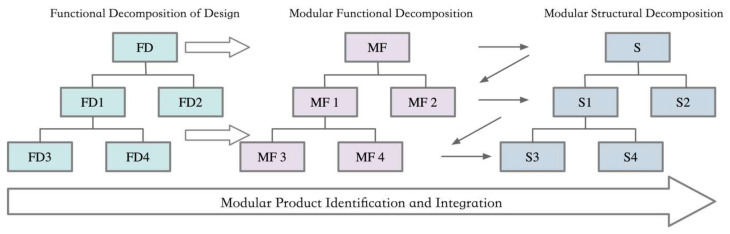
Modular product composition process.

The process of modular product identification and integration involves the visual representation of the product system. Its core lies in determining the scope of module identification based on the degree of coupling between elements [32]. Through in-depth functional analysis, we can identify and design a series of functionally, performatively, and structurally distinct modules that are interchangeable and independently structured. By carefully selecting and combining these modules, products with varying performances and specifications can be created. The visual representation of the product’s functional structure, as shown in the structural diagram, is a critical step in the module identification process. This step requires a clear presentation of information, including the derivation of information from left to right and the identification and merging process of “Z”-shaped mapping, as shown in Figure 3.

### 2.2. Perspectives and Principles of Module Extraction

Modules are the fundamental units that make up complex systems [33]. The method of module extraction not only determines the granularity of the modules but also affects the richness of the attribute information contained within them. The choice of extraction method has a significant impact on the system’s layout, performance, and structure. Therefore, reasonable module extraction is a critical prerequisite for ensuring the smooth progression of subsequent layout planning. Module extraction can be either coarse or detailed, with the key being to avoid having too many or too few modules. Having too many modules, while beneficial for assembling a diverse range of products, increases workload and complexity. On the other hand, having too few modules reduces the workload but diminishes the generality of the modules, making it difficult to expand the range of product types and potentially failing to meet specific product requirements. Therefore, selecting the appropriate perspective and method for module extraction and establishing clear extraction principles are crucial factors in ensuring the rationality of the module extraction process.

**Figure 4:**
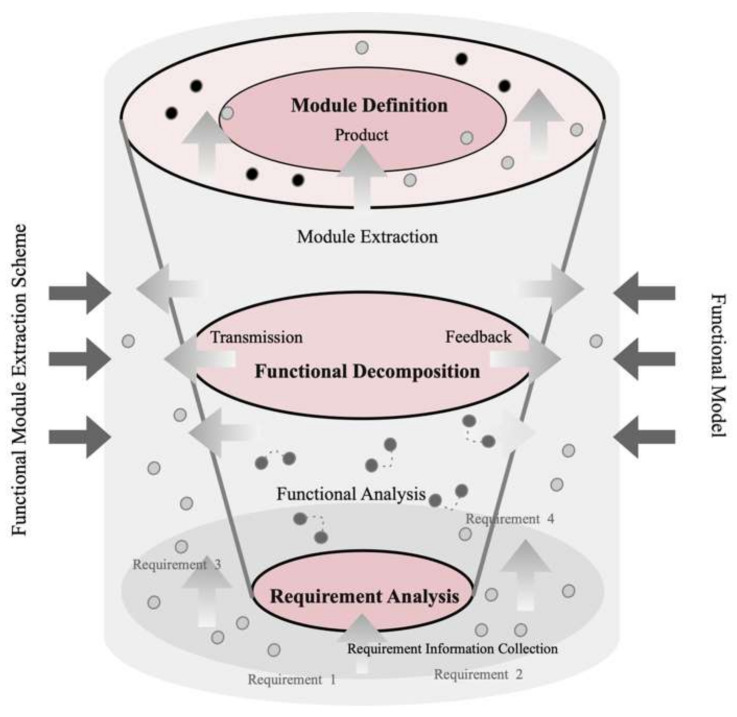
Module extraction process.

The results of module extraction can vary depending on the perspective taken. This is a top-down process that encompasses five stages: requirements analysis, functional analysis, functional decomposition, module extraction, and module definition. The process begins with the collection of requirement information and, through systematic functional analysis and decomposition, gradually refines the complex system into manageable sub-functions, ultimately identifying and defining independent modules. Each stage is closely interconnected through transmission and feedback mechanisms, ensuring that the design continuously optimizes while meeting user requirements, and maintaining systematization and adaptability in the design. This precise and structured design method is particularly well-suited for the development of complex products, significantly improving design efficiency and product quality, as shown in Figure 4.

### 2.3. Study of Module Mapping Relationships

**Figure 5:**
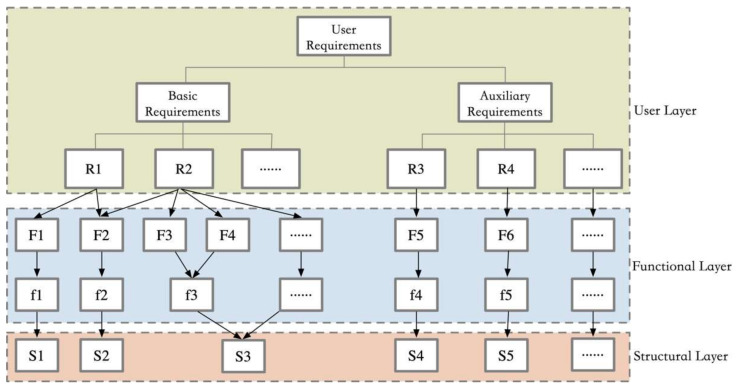
Hierarchical division of module identification.

The piano practice assistance device, as a complex product system, is composed of numerous highly coupled system modules, many of which have multiple structural layers and are themselves quite complex products or systems [34]. The process of module identification in complex product systems requires a structured approach to decomposition [35]. Through the analysis of the vertical structure, the various levels of complex product modules can be clearly articulated. After the vertical structure model is gradually established, it needs to be further refined. During the hierarchical study process, the primary and secondary user needs should be identified as independent modules. The identification of the product hierarchy is achieved through the mapping between needs and functions, followed by the design of a series of structural modules to realize the product functions, thereby meeting user needs. By designing a structure matrix, closely related structures are grouped together to form functional structure modules. The user layer, function layer, and structure layer are the primary modules for product hierarchy identification, with their mapping relationships illustrated in Figure 5. 

**Figure 6:**
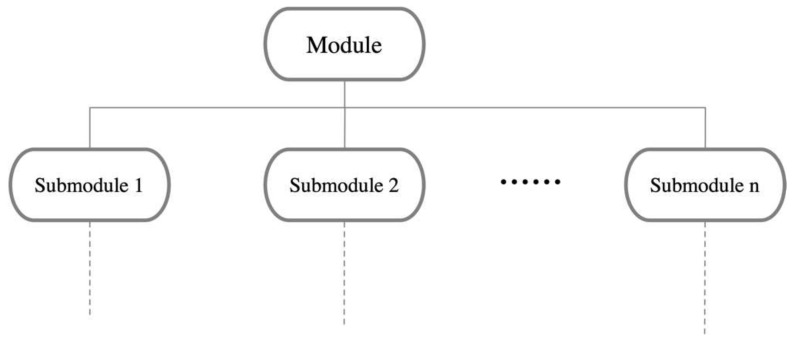
Hierarchical structure tree of modules.

The core significance of modular design lies in decomposing the same product into different modules, which can then be reorganized or derived to design new product modules [36]. This approach, compared to traditional design, is more adept at meeting the diverse needs of different user groups and facilitating personalized customization. As this emerging model gains traction, modularization has become an indispensable component and is increasingly recognized and endorsed by users. The organizational structure of piano practice assistance devices is relatively complex, and due to the intricacies of user needs, structural composition, and technical conditions, it is essential to achieve scientific and efficient module identification. The core idea of modular identification for complex products is to break these products down into a series of modules and integrate the product structure through the configuration and connection of these modules. By constructing a product system architecture, we create an abstract hierarchical product structure that reflects the process of gradually clarifying from an initial vague understanding of how to meet user needs to a clear and specific structure. The product system architecture can be expressed in various ways, with the hierarchical structure tree being a widely used method. This approach not only clearly demonstrates the components of the product but also helps in understanding the relationships and interactions between the various modules, as shown in Figure 6.

**Figure 7:**
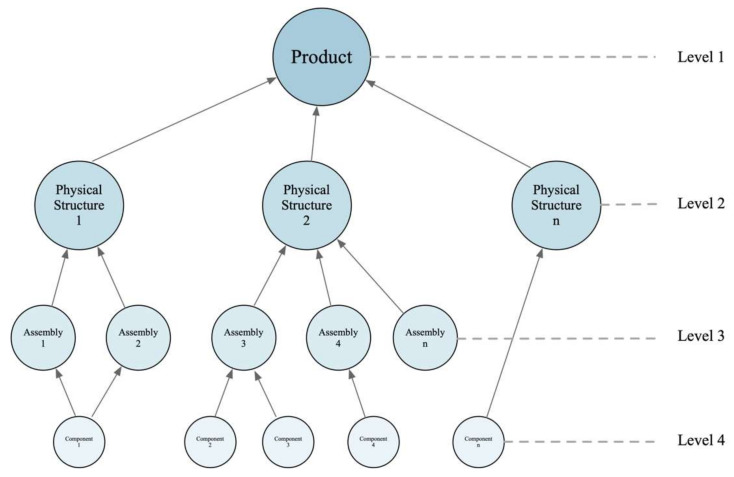
Product hierarchical structure tree.

Establishing a bottom-up product module architecture, the process of product module identification can be understood as transitioning from product components to assemblies and then to the product’s physical structure. This approach can be viewed as a reverse thinking process for solving problems, as illustrated in Figure 7.

### 2.4. Functional Model Decomposition

One of the effective strategies for solving complex design problems is to use a hierarchical approach, which involves decomposing and simplifying the problem, then refining it step by step from a higher level of abstraction downwards [37]. This method, which transitions from abstract to concrete and from whole to part, is not only an effective means of problem-solving but also aids in gaining a deeper understanding of the essence of things. In product design, the precise visualization of functions is crucial, especially for those functions that cannot be directly mapped to specific behaviors or structures. Functional decomposition allows the overall function to be subdivided into smaller sub-functions, with each sub-function maintaining clear logical and physical relationships with the others.

**Figure 8:**
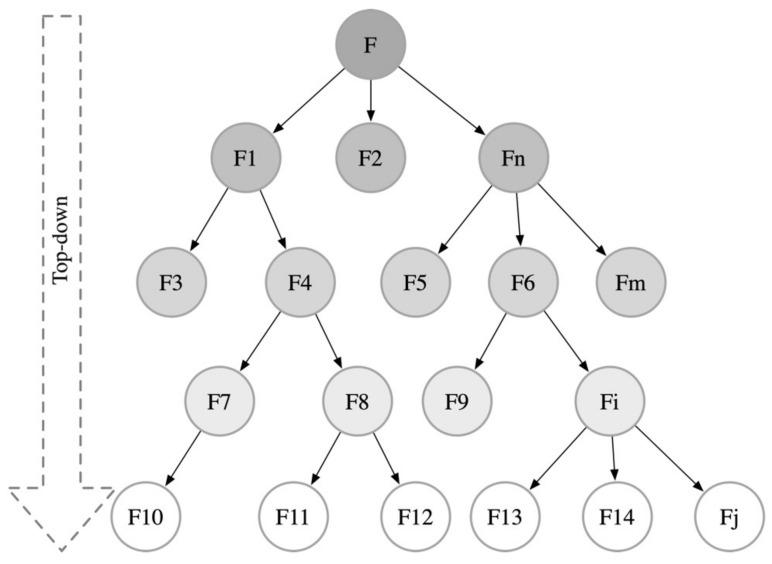
Functional method tree.

The details of functional decomposition, such as the number of layers and the number of sub-functions within each layer, should be determined based on the novelty of the task and the depth of analysis required for the sub-functions. During the initial stages of design, designers may not have a complete understanding of all functional elements or may be unable to accurately predict the optimal combination of functional elements. Therefore, finding and establishing an optimal functional structure becomes crucial during the conceptual design phase. The decomposition of the functional model is a process of identifying and optimizing the configuration of product sub-functions based on specific rules and constraints. This process ensures that the overall function of the product can be realized through an orderly hierarchical structure of its sub-functions. As such, constructing a functional method tree is a key step, allowing designers to perform a detailed, granular decomposition of the functional model down to the level of specific functional elements. This precise functional decomposition process not only helps clarify design goals and requirements but also promotes the systematization and structuring of design thinking, enabling designers to more effectively develop products that meet user needs and technical specifications. This design approach highlights the central role of functional decomposition in helping designers thoroughly understand product complexity and achieve design innovation, as illustrated in Figure 8.

In the functional method tree, the branches are referred to as functional elements, which are the smallest constituent units of the functional structure. The decomposition of the functional method tree concludes when the smallest functional modules have been identified. The functional method tree, as a standardized system for expressing functional decomposition, is a key step in constructing a functional semantic network. By utilizing the functional model decomposition process to express and analyze conceptual knowledge, this process facilitates the reorganization of knowledge and the development of module configurations, while also providing the necessary constraints for constructing the principle model.

In design practice, designers need to decompose the required functional hierarchy into multiple sub-functions, which gradually become more concrete within the hierarchical structure [30]. This strategy not only supports the solution of conceptual design but also leverages knowledge representation to articulate product design schemes, thereby allowing users to describe functions flexibly. Throughout the design process, functions are progressively transformed into specific behavior and structural models. This transformation aids in moving from theoretical concepts to practical implementation, ensuring the accurate realization of design intent and the fulfillment of functional requirements.

### 2.5. Product Functional Organizational Structure Modeling

**Figure 9:**
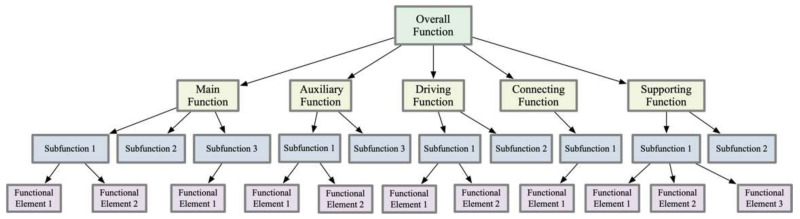
Product functional structure tree.

**Figure 10:**
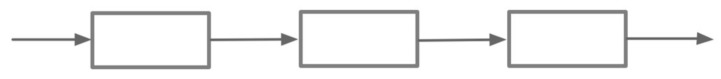
Transformation and transmission between functional elements.

**Figure 11:**
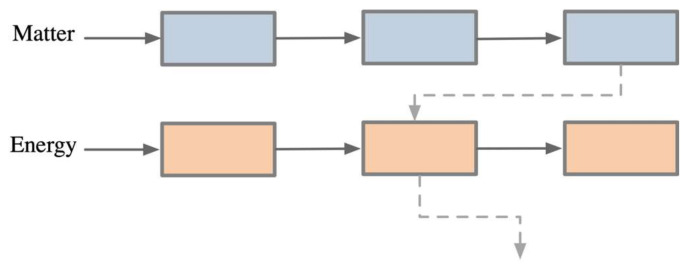
Product functional structure diagram.

The essence of product functional innovation lies in the iterative optimization of the functional organizational structure [38]. Product functional organizational structure modeling is a crucial step in the design modeling process and represents the final form of the product’s functional semantic network. It provides a reference for the construction of the principle model. The specific steps for product functional organizational structure modeling are as follows:

(1) Clarify the Overall Design Objectives: Establish the overall function based on user needs and provide a qualitative description of the overall function.

(2) Quantitative Description of the Overall Function: Clarify the internal relationships between functions and perform hierarchical mapping within the overall function to form a product functional decomposition tree. The tree should clearly depict the hierarchical relationships between product functions in a branching format. The overall function is divided into a series of sub-functional modules based on their attributes, and these sub-functions are further decomposed to generate functional elements, as illustrated in Figure 9.

(3) Establish the Matching Relationships Between Functions: Form a complete functional chain through the input-output relationships, creating a left-to-right functional model matching scheme, as illustrated in Figure 10.

(4) Establish the Functional Structure: To avoid the creation of excessive redundant functional elements, it is necessary to merge overlapping parts while establishing the functional structure. This ensures that each functional unit can be mapped to the lower-level product modules, as illustrated in Figure 11.

**Figure 12:**
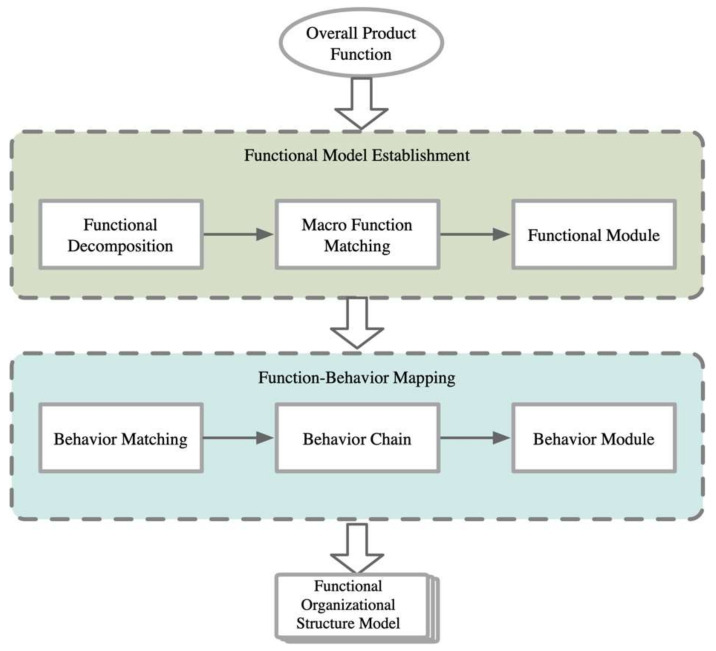
Functional organizational structure modeling process.

Product functional organizational structure modeling is a crucial part of functional modeling and solution development. It involves establishing functional modules through functional decomposition, macro-function matching, and mapping functions to behaviors, all based on the overall product function. This process culminates in the creation of the product’s functional organizational structure model, as illustrated in Figure 12.

## 3. Top-Down Product Design Process

### 3.1. Top-Down Multi-Level Assembly Model Construction

**Figure 13:**
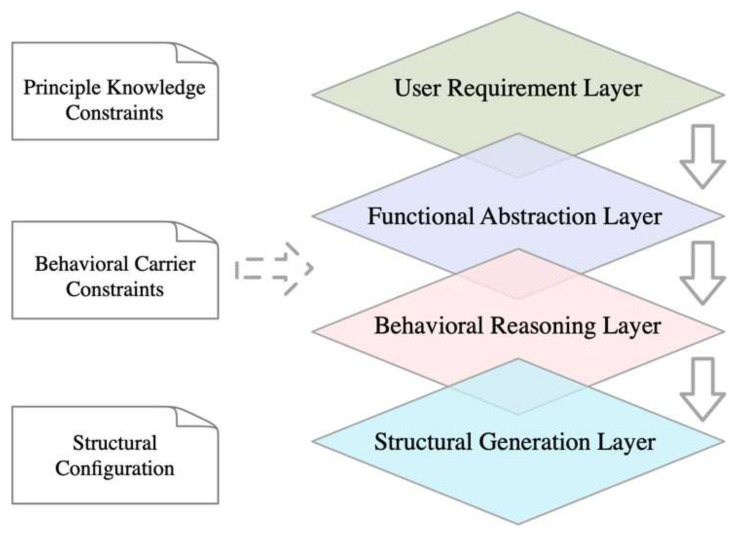
Top-down process model.

The top-down process model is a typical approach for solving the mapping of product functional structures [39]. In this process, the first step is to analyze the user requirements for the product to be designed, followed by an abstract representation of the product’s overall function. Next, this overall function is decomposed layer by layer until it reaches the most basic supporting functions, which are usually already in existence or relatively easy to achieve. Through multi-level module mapping, the required structural modules can be constructed. The top-down design process begins with determining the product’s hierarchical structure, forming an organized structural system. An envelope is then created around this structural system, using elements like reference features to establish constraint relationships between the various component structures. Under the overall constraint requirements, each component structure is designed in detail, ensuring that when making overall adjustments, the entire product and its components can be managed from the top level. As illustrated in Figure 13, this process not only ensures the systematization and consistency of the design but also enhances control over the design details of the product.

In the top-down modular design process for a piano practice assistance device, the process includes the user needs layer, functional abstraction layer, behavior reasoning layer, and structural generation layer [40]. This is a mapping process that transitions from abstract concepts to concrete implementations. During this process, the granularity of functional decomposition is gradually refined, resulting in a scientifically sound and reasonable module design solution through principle knowledge constraints and behavior carrier constraints. To organize disordered design information, it is essential to establish a structured information architecture. The creation of this information structure should always be guided by user needs, clearly defining the module content required at each design step. By continuously breaking down and aggregating information, the solution for the product’s underlying structural modules is achieved.

**Figure 14:**
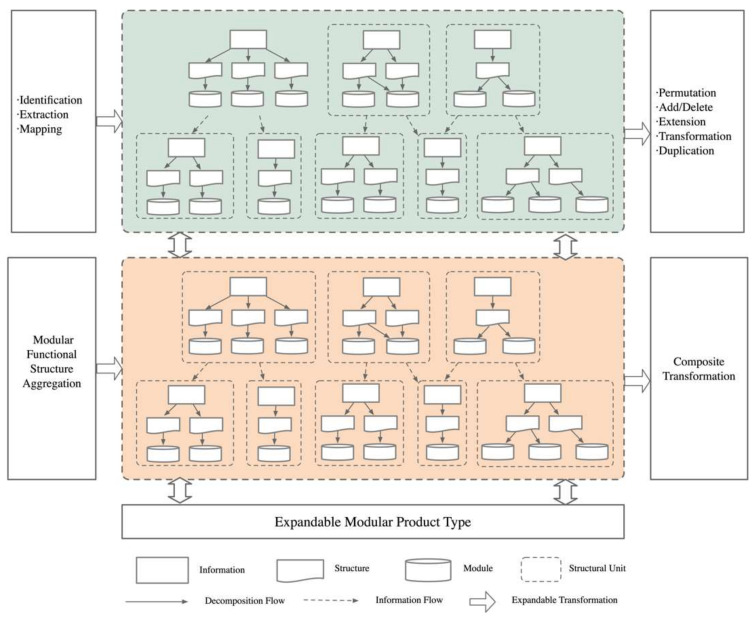
Module variant design strategy.

By establishing an information flow, this study optimized the transfer process between structural units and decomposed the functional structure model, effectively reducing the coupling between modules. Based on this, the application of extension transformation to the functional structure design decomposition model enabled the aggregation of module functional structures and the construction of the desired module’s functional structure decomposition model. Additionally, composite transformations of related structural units facilitated the integration of the product structure. In the development process of mechanical products, the first step is to analyze market demands and identify the functional components required for the new product. Based on these needs, an initial design principle plan is formulated, constructing a conceptual composition model of the product. This conceptual model is then analyzed and calculated to obtain the assembly model. Using the design information from the assembly model, a detailed design of the subassemblies is completed, followed by analysis and adjustments to the design until a mechanical product that meets the functional requirements is achieved. This top-down design process not only aligns with design logic and the forward-thinking approach of designers but also facilitates parallel design of subassemblies or components in later stages. This method allows the establishment of key information at the highest level of product design, enabling modifications and extended designs of the product, ensuring overall structural rationality. It is particularly suitable for the development and adjustment of mechanical products. During this design modeling process, the extensibility of the product critically determines the level of innovation, demonstrating the effectiveness and innovativeness of the top-down multi-level assembly model construction, as illustrated in Figure 14.

### 3.2. Product Platform Establishment

**Figure 15:**
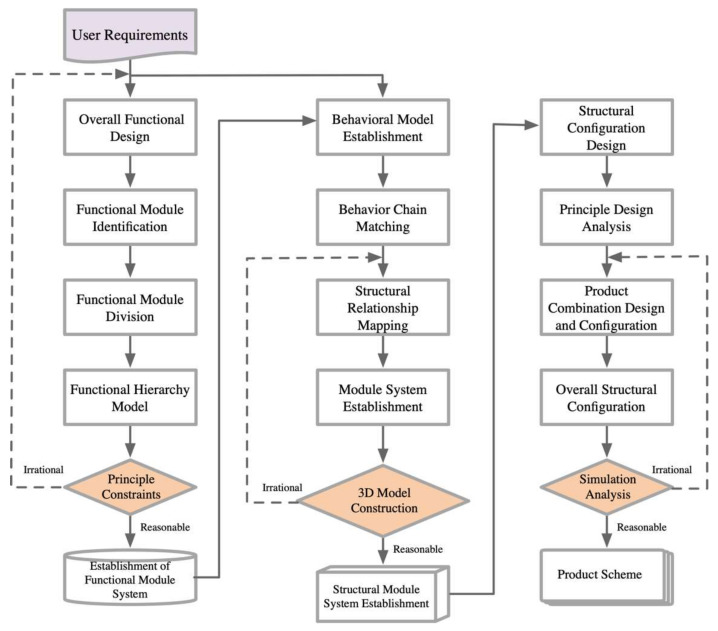
Product platform establishment.

The establishment of a piano practice assistance device platform is a multi-layered combinatorial optimization process that involves the systematic reconstruction of various modules to achieve a design solution that meets user needs. This process begins with the analysis and categorization of user needs, followed by the creation of a user needs module that maps these needs to corresponding functions. Subsequently, these functions are organized into a structural model and mapped to principle models, which serve as constraints for building behavior models. Once this step is complete, the process moves to mapping from behavior to structure. If, during this process, it is found that the functional modules do not reasonably align with the principle models, the product functional modules need to be restructured. After the structural mapping is completed, the next step is to configure and solve the product modules. The evaluation of the product configuration solution is then carried out through simulation analysis. The overall design process and the evaluation results of this design scheme are illustrated in Figure 15.

## 4. Construction of the Assembly Model for Piano Practice Assistance Mechanisms

### 4.1. Target Population and Needs for Wearable Exoskeleton Piano Aids

The wearable exoskeleton piano practice aid is primarily designed for piano learners with neurological disorders, particularly beginners. This aid automatically adjusts the hand to the correct key positions, with the main purpose of assisting in correcting hand positioning and improving playing accuracy, thereby enhancing practice efficiency and ensuring effective training. Through consistent and correct practice, learners can achieve their learning goals. Incorrect hand positioning or emotional instability can distract learners, reduce self-control, and negatively impact their psychological and physiological states. Piano playing involves not only artistic expression but also various mental activities such as thinking, memory, creativity, and emotional fluctuations, all of which are crucial to practice efficiency and performance quality.

To precisely meet the needs of this target group, we conducted a series of research activities, including interviews with 70 patients and literature reviews covering conditions such as Parkinson’s disease, multiple sclerosis, and amyotrophic lateral sclerosis (ALS). Through expert discussions and feedback from user focus groups, atypical needs were excluded, and a questionnaire was developed using a 5-point Likert scale to assess the importance of various functions. Based on this data, we calculated the weight of user needs, which then guided the product design. The user needs and their corresponding weights (K values) were clearly documented to ensure that the design aligns with the true intentions of the users, as shown in Table 1.

### 4.2. Piano Practice Aid Functional Module Extraction

Based on an in-depth analysis of the target population’s needs, this study develops a multifunctional wearable exoskeleton piano practice assistance device, integrating key functions such as finger shape correction, automatic control, mobile positioning, stability support, and power drive through systematic design. The design of the device is based on a multi-domain mapping approach, integrating three aspects: the functional domain, the structural domain, and the user needs domain, to ensure the scientific nature of function realization, the rationality of structural design, and the efficiency of user experience.

At the functional domain level, the device is designed to achieve core functions such as hand shape correction, key position guidance, and force feedback, meeting the special needs of neurological rehabilitation patients during the piano learning process. The finger shape correction function is accomplished through a main thumb correction component and four auxiliary finger correction components working in tandem. The main thumb component ensures the accuracy of thumb positioning, while the remaining components make precise adjustments to the other fingers. Additionally, the device uses five push rod motors to drive the hand support system, enabling automatic guidance and positioning of the user’s wrist, ensuring that the fingers accurately strike the keys and maintain an ideal posture during performance.

At the structural domain level, the device relies on a physical structure built through multi-module collaboration. The control module is electrically connected to the bracket assembly and power devices for precise automated control. The bracket assembly consists of arc-shaped and inverted T-shaped components, forming an inverted T-shaped sliding groove, ensuring stability in support and flexibility in movement. The driving function is realized through transverse motors and driven wheels, working with the link mechanism of the hand support system to simulate the natural key-pressing movements of the human hand.

**Figure 16:**
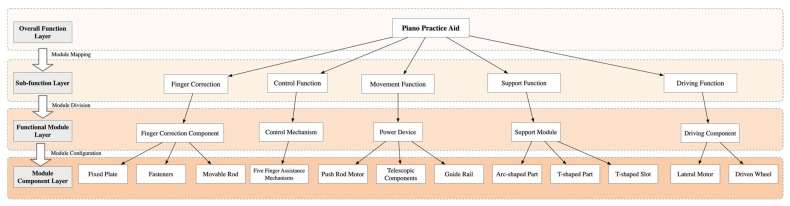
Functional module tree of the piano practice assistance device.

At the user needs domain level, the device design fully considers the practical needs of neurological rehabilitation patients and beginners, such as ease of operation, correction accuracy, and comfort. The application of the multi-domain mapping approach efficiently maps functional requirements to physical modules, creating a targeted and structurally optimized design solution. This not only significantly enhances the adaptability and flexibility of the device but also provides a new path for the scientific design of piano practice assistance devices. By integrating the core concepts of multi-domain mapping, combining functional requirements, structural realization, and user needs, the device ensures diversity and reliability in its performance, offering an innovative solution for piano learning and rehabilitation, as shown in Figure 16.

### 4.3. Establishment of the Functional and Structural Module Mapping Library

For functional modules, this study proposes a wearable exoskeleton piano practice assistance device based on multi-domain mapping. Through the mapping of functional modules to structural modules, key functions such as finger shape correction, key position guidance, and automation control are organically integrated. The aim is to address issues that piano learners may face during practice, such as fatigue, attention deficits, and difficulties in finger shape correction. The device consists of four major modules: the hand support system, power device, bracket assembly, and drive components. The corresponding relationships between their functions and structures are shown in Table 2. The hand support system works in coordination with the frame and five-finger assistance mechanisms to fix the user’s hand back. It uses hinged structures with L-type linkages, a first linkage, and a second linkage to enable precise finger pressing on the piano keys. The power device is composed of five push rod motors, whose rods are connected to the L-type linkages of the finger assistance mechanisms, driving each finger to independently press the keys. The bracket assembly includes extendable components and guide rails. Through an adjustable structure of the first and second tubes, it guides the user’s wrist along the length of the piano keyboard to the correct tonal area. The drive components consist of lateral motors and driven wheels. The lateral motor moves the hand, while the driven wheels assist in switching the user’s hands between different tonal areas. In practical use, the control module moves the user’s wrist to the target tonal area while driving the finger assistance mechanism to press the target key and monitor and correct the finger shape in real-time to ensure the user maintains the ideal posture during each practice session.

The device combines modular design with the core concept of multi-domain mapping, starting from the functional, structural, and user needs domains, to achieve efficient mapping and execution of functional requirements to physical structures [41,42]. In actual practice scenarios, the user’s hand is placed in the bracket assembly. Once the device is activated, the bracket assembly works in tandem with the power device to move the wrist to the target tonal area. The push rod motors drive the finger assistance mechanisms to complete the key pressing actions. This design not only ensures the accuracy of key presses but also optimizes the user’s learning experience through real-time finger shape correction. Through multi-dimensional functional module integration and structural optimization, this device significantly enhances the user’s practice efficiency and action accuracy while addressing the high demands for finger posture and movement coordination in piano practice. It provides innovative technical support for piano teaching and neurological rehabilitation fields, fully demonstrating the value of multi-domain mapping design in complex systems.

With the wearable exoskeleton piano practice aid, piano learners can improve their hand positioning during practice, facilitating the formation of muscle memory and the development of unconscious key-pressing and control habits. The device includes a bracket assembly with retractable components and guides that direct the learner’s wrist along the piano keyboard to the correct musical zone. This assistance not only provides immediate benefits with each use but also has a lasting impact on various aspects of piano performance. Additionally, the wearable exoskeleton piano practice aid subtly corrects the learner’s hand positioning at a subconscious level through its unique structure. Each correction is adjusted to align with different practice stages and hand positioning requirements, catering to the specific needs of various pieces of music. The design takes into account the performance structure and technical demands of the repertoire, making hand positioning corrections more targeted. Through this multi-channel and multi-dimensional visual and tactile experience, the device helps learners adapt to hand positioning changes across different pieces, ensuring the effectiveness and adaptability of practice.

### 4.4. Simulation Analysis of the Piano Exoskeleton Practice Aid

The product designed in this study is a wearable exoskeleton piano practice aid, specifically designed to automatically guide the piano learner’s hand to accurately move to the designated musical range and assist the fingers in precisely pressing the keys while maintaining the ideal finger posture throughout the process to achieve active hand positioning correction. This device not only automatically positions the hand in the correct musical zone and helps the fingers press the appropriate keys but also ensures that the fingers maintain the correct posture during playing. This greatly reduces the learning difficulty for beginner piano students and effectively decreases fatigue caused by long practice sessions. The key components of the device include a bracket assembly, at least one hand assistance system, a power unit, and a control module. The bracket assembly is responsible for moving the wrist along the length of the piano keyboard to the corresponding musical zone. The hand assistance system consists of a frame and five-finger assistance mechanisms corresponding to the five fingers, which are driven by the power unit through hinged connections to press the keys. To ensure that the design meets the specific needs of the users, we will conduct a degree of freedom analysis and workspace analysis of the piano practice aid mechanism.

#### 4.4.1. Degrees of Freedom

**Figure 17:**
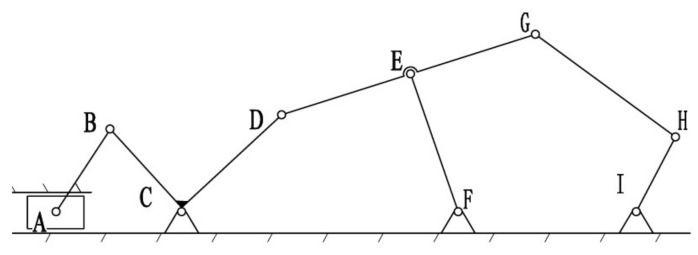
Simplified diagram of the finger mechanism.

Based on the designed mechanical hand, considering one of the fingers, we can treat a single finger as a planar linkage mechanism. The mechanism can be simplified, and the simplified kinematic diagram of the mechanism is shown in Figure 17.

Formula for Calculating the Degrees of Freedom of a Planar Linkage Mechanism

(1) $$F=3n-2p_{l}-p_{h}.$$

In the formula:

n: Represents the number of moving links in the mechanism; in this mechanism, the number is 5.

$p_{l}$: Represents the number of lower pair constraints in the mechanism; in this mechanism, the number is 7.

$p_{h}$: Represents the number of higher pair constraints in the mechanism; in this mechanism, the number is 0.

(2) F=3×7−2×10−0=1.

Through calculation, it is determined that the mechanism has one degree of freedom, meaning the end of the mechanism possesses one degree of freedom, represented by HI for the finger part. In designing the driving section, we design one driver for each finger and installed it on the wrist. The movement of the A slider, which corresponds to the reciprocal motion of the electric push rod, drives the mechanism, while the other parts follow accordingly.

#### 4.4.2. Kinematic Analysis

**Figure 18:**
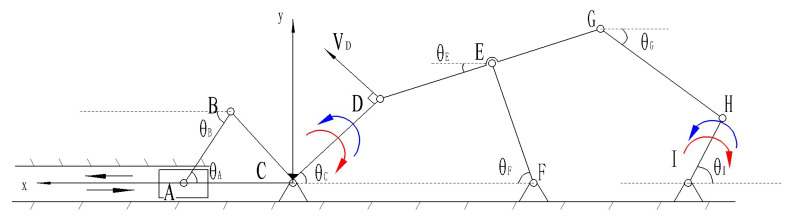
Simplified mechanism motion state analysis diagram.

The velocity analysis is illustrated in Figure 18.

Through the analysis of the mechanism’s motion, when the slider moves to the left, the rod rotates counterclockwise (in the direction of the blue arrow), and when the slider moves to the right, the rod rotates clockwise (in the direction of the red arrow). To analyze the velocity of point G, it is understood that this mechanism is a compound linkage mechanism composed of a left-side crank–slider mechanism + a right-side double rocker mechanism + a five-bar linkage mechanism. Therefore, the velocity calculation is divided into two parts.

Included among these, $\left|V_{B}\right|=\left|V_{D}\right|$, $\left|a_{B}\right|=\left|a_{D}\right|$.

Let the velocity at point A be $V_{A}$ and the magnitude of the applied force F is $V_{By}=V_{A}\cos\theta_{A}\sin\theta_{A}$.

Based on the relationships depicted in the above diagram, the following can be derived:

(3) $$V_{AB}=V_{A}\cos\theta_{A},V_{Bx}=V_{A}\cos^{2}\theta_{A},V_{By}=V_{A}\cos\theta_{A}\sin\theta_{A}$$

Therefore, according to the Pythagorean theorem, it can be derived that

(4) $$V_{B}=\sqrt{V_{Bx}^{2}+V_{By}^{2}}=V_{A}\cos\theta_{A}.$$

The direction is perpendicular to BC;

Similarly, for, $V_{D}=V_{A}{\cos\theta }_{A},\mathrm{The}\;\mathrm{direction}\;\mathrm{is}\;\mathrm{perpendicular}\;\mathrm{to}⊥\mathrm{CD}$.

**Figure 19:**
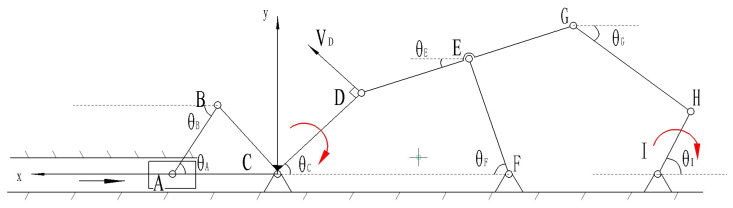
Simplified diagram of the unidirectional motion analysis of the mechanism.

Next, analyze the right half, as shown in Figure 19.

CDEFC is a double-rocker mechanism. When the slider moves to the right, the vector equation can be derived from the closed vector loop relationship.

(5) $$\vec{l_{CD}}+\vec{l_{DE}}=\vec{l_{CF}}-\vec{l_{EF}}$$

The complex vector form is given by

(6) $$l_{CD}e^{i\theta_{C}}+l_{DE}e^{i\theta_{E}}=l_{EF}e^{i\theta_{F}}+l_{CF}.$$

By differentiating, we obtain:

(7) $$\left\{\begin{array}{l} l_{CD}\cdot \omega_{DC}\cdot \cos\theta_{C}+l_{DE}\cdot \omega_{DE}\cdot \cos\theta_{E}=l_{FE}\cdot \omega_{EF}\cdot \cos\theta_{F}\\ l_{CD}\cdot \omega_{DC}\cdot \sin\theta_{C}+l_{DE}\cdot \omega_{DE}\cdot \sin\theta_{E}=l_{FE}\cdot \omega_{FE}\cdot \sin\theta_{F} \end{array}\right\},$$

where $\omega_{DC}=\frac{V_{D}}{l_{CD}}$ represents the magnitude, and the direction is clockwise.

Let the unknowns $\omega_{DE},\;\omega_{FE}$ be represented by x,y, respectively.

Then, we have

(8) $$\left\{\begin{array}{l} l_{FE}\cdot \cos\theta_{F}\cdot y-l_{DE}\cdot \cos\theta_{E}\cdot x=V_{A}\cos\theta_{A}\cos\theta_{F}\\ l_{FE}\cdot \sin\theta_{F}\cdot y-l_{DE}\cdot \sin\theta_{E}\cdot x=V_{A}\sin\theta_{A}\sin\theta_{F} \end{array}\right\}.$$

Using Cramer’s rule to find the general solution,

the prerequisite is

(9) $$l_{FE}\cdot \cos\theta_{F}\cdot \left(-l_{DE}\cdot \sin\theta_{E}\right)\neq l_{FE}\cdot \sin\theta_{F}\cdot \left(-l_{DE}\cdot \cos\theta_{E}\right).$$

The solution is obtained as:

(10) $$\omega_{DE}=\frac{l_{DE}\cdot \cos\theta_{E}\cdot V_{A}\sin\theta_{A}\sin\theta_{F}+l_{FE}\sin\theta_{F}\cdot V_{A}\cos\theta_{A}\cos\theta_{F}}{l_{FE}\cdot \cos\theta_{F}\cdot l_{DE}\sin\theta_{E}-l_{DE}\cdot \cos\theta_{E}\cdot l_{FE}\sin\theta_{F}}.$$

The direction is rotational around point E.

Therefore, it can be concluded that

(11) $$V_{G}=\omega_{DE}\cdot l_{EG}=\frac{l_{DE}\cdot \cos\theta_{E}\cdot V_{A}\sin\theta_{A}\sin\theta_{F}+l_{FE}\sin\theta_{F}\cdot V_{A}\cos\theta_{A}\cos\theta_{F}}{l_{FE}\cdot \cos\theta_{F}\cdot l_{DE}\sin\theta_{E}-l_{DE}\cdot \cos\theta_{E}\cdot l_{FE}\sin\theta_{F}}\cdot l_{EG}.$$

#### 4.4.3. Workspace

**Figure 20:**
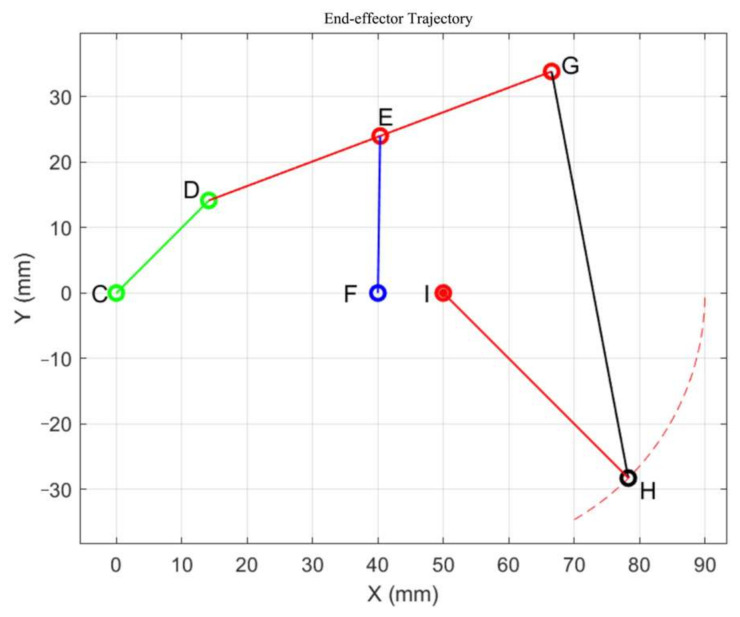
Workspace solution diagram.

After determining the motion of the left half of the L-shaped link, the right end of the L-shaped link can be regarded as a combination of a crank–rocker mechanism and a five-bar mechanism. The workspace model is drawn using MATLAB tools, as shown in the figure below (Figure 20). The motion trajectory of point H is found to be similar to the movement of the proximal segment of a human finger. The lengths of the various links are as follows:

(12) $$l_{CD}=20\mathrm{mm},\;l_{DG}=48\mathrm{mm},\;l_{EF}=28\mathrm{mm},\;l_{GH}=60\mathrm{mm},\;l_{IH}=40.$$

By inputting the lengths and positions of each linkage, programming the calculations, and setting the constraints (i.e., the rotational range of segment CD at the right end of the L-shaped linkage and the length of each linkage), the workspace is computed as shown in Figure 20. Systematic optimization of each finger module and analysis of the joints at the proximal end of each finger can facilitate piano playing. In the same environment, the bending angles of each exoskeleton joint unit were derived. As shown in Figure 20, the dashed line represents the movement trajectory of the finger’s proximal end, with a bending angle of approximately 60°. Consequently, it can be concluded that 60° is the maximum bending angle of the aforementioned joint, slightly less than the maximum bending angle of a human hand, thereby verifying the scientific validity of the finger assistance mechanism design.

### 4.5. Wearable Exoskeleton Hardware Product Model

**Figure 21:**
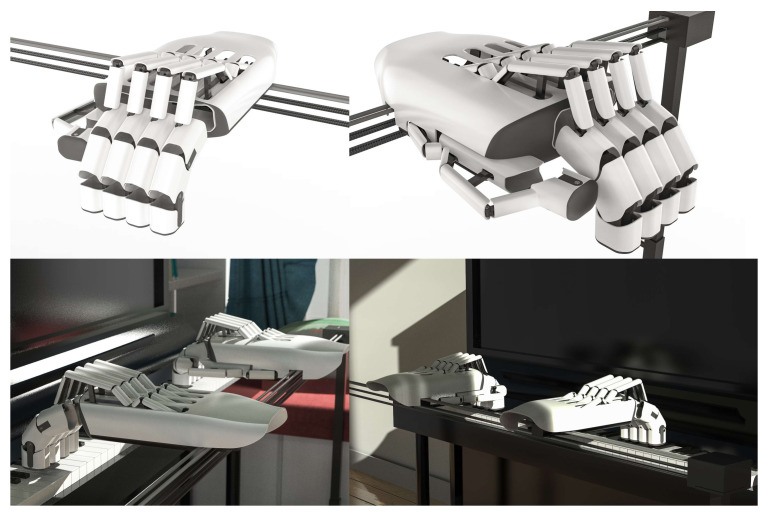
Piano practice aid layout and structural morphology diagram.

Based on the aforementioned functional analysis and simulation, we have designed and developed an innovative piano practice aid using wearable exoskeleton technology aimed at enhancing the efficiency and precision of piano learning. This device is equipped with advanced driving mechanisms and hand assistance systems, specifically for dynamic kinematic correction and real-time finger adjustment. The application of these technologies not only ensures the accurate performance of musical scores but also significantly reduces psychological stress during practice while improving learning motivation and efficiency. Part of the research also includes productization modeling of the device, as shown in Figure 21, with detailed information presented in the relevant diagrams, further confirming its practicality and application potential. Additionally, the design of the wearable exoskeleton piano aid takes into account human physiological structures, allowing it to coordinate movement with the user’s hand. It primarily consists of a bracket component, a power module, a control unit, and a hand assistance system. The bracket component guides the wrist along the piano’s length to specific octaves, while the power module drives the hand assistance system to press the keys. This aid supports automated operation, further enhancing the efficiency and level of piano learning.

## 5. Discussion

This study adopts a combination of top-down design processes and layout constraints to systematically construct a model of the piano rehabilitation assistance device. Through tree diagram specialization and multi-body system low-order array technology, the topological relationships, assembly sequences, and spatial positions of the layout structure modules are clearly defined. The mapping between functional modules and structural modules is established, and a complete three-dimensional model is further formed. Simulation analysis results validate the rationality and practicality of the design, which not only optimizes the design process but also provides a reliable theoretical and practical foundation for the modular design and system integration of complex devices. Based on this, this study proposes a wearable exoskeleton piano practice assistance device based on multi-domain mapping and a top-down process model, significantly enhancing its modular design and functional coordination by integrating the functional domain, structural domain, and user needs domain.

Compared to existing research, this device demonstrates significant advantages in functional module integration and motion precision optimization. For example, compared with the soft robotic glove developed by Florida Atlantic University [15], this device not only provides mechanical assistance and finger shape correction but also achieves higher motion precision and continuity through a modular drive design. Its real-time finger shape correction function compensates for the shortcomings of traditional devices in long-term posture maintenance. Compared to “The Piano Press Glove” [16], this device also incorporates wrist positioning and key position guidance functions, further enhancing the user’s self-practice efficiency and experience. The innovation of this study lies in achieving precise motion allocation and module functional coordination through multi-domain mapping, optimizing high-precision control of key press actions by combining push rod motors with finger assistance mechanisms, and using modular design to flexibly configure the device according to user needs, enabling high customization [43,44], which significantly enhances the device’s adaptability and practicality in piano teaching and neurological rehabilitation.

This device has significant application value in both the rehabilitation and music education fields. In the rehabilitation field, the device supports the restoration of finger function for neurological rehabilitation patients through finger shape correction and key position guidance functions. Simulation analysis results show that its motion precision highly aligns with the user’s biomechanical characteristics, significantly improving the scientific effectiveness of rehabilitation training. In the music education field, the device addresses common issues faced by beginners, such as incorrect finger shapes and uncoordinated movements, through the integration of multifunctional modules. The device reduces the need for teacher intervention in finger correction, providing intuitive, dynamic learning support for beginners while effectively reducing learning fatigue. Additionally, the device’s innovative design combines piano teaching and neurological rehabilitation, offering a new path for the integration of educational and medical technologies.

Despite significant progress, this study still has limitations, including a small sample size and insufficient depth in experimental comparative analysis. Future research can further optimize the following directions: expanding the sample size, particularly for validation studies targeting different age groups and patients with neurological diseases; developing more flexible control algorithms to adapt to diverse usage environments; and exploring the integration with virtual reality (VR) technology to create immersive practice scenarios, enhancing the interactivity and fun of the device. These research directions are expected to further improve the device’s technical level and practical application value, providing more comprehensive solutions for the music education and neurological rehabilitation fields.

## 6. Conclusions

This study developed an innovative wearable exoskeleton piano practice assistance device that adopts a modular design concept, combines ontology theory with multi-domain mapping methods, and proposes a top-down design process, effectively achieving the efficient conversion from functional requirements to structural implementation [41,45]. Through strategies such as module identification, extraction, and mapping, the device demonstrates significant technical advantages in finger shape correction, key guidance, and motion precision optimization. Test results indicate that the device shows high motion consistency and biomechanical compatibility in both simulation and practical verification, providing strong support for the scientific and reliable performance of the device.

The research results show that the device not only achieves a high level of motion precision and stability but also significantly optimizes the user practice experience, opening new pathways for the integrated application of music education and neurological rehabilitation. The modular design and flexible configuration of the device enable it to meet the needs of different usage scenarios and target users, particularly offering unique advantages in guiding the finger shape of beginners and restoring hand function for rehabilitation patients. By integrating multifunctional modules, the device effectively overcomes the limitations of traditional piano practice assistance devices in terms of flexibility, applicability, and long-term effectiveness. The continued deepening of this research is expected to promote the widespread application of intelligent assistive devices in music education and rehabilitation therapy, providing more innovative solutions and practical value for the development of this field.

## Figures and Tables

**Table 1:** User needs weight analysis.

User Requirement P.	Requirement Weight K.
Finger Position Correction	0.209
Practice Assistance	0.2239
Free Movement	0.1891
Strong Support	0.199
Aesthetic Design	0.1791

**Table 2:** Functional module to structural module mapping library.

Functional Module		Corresponding Structural Module	Structural Description	Functional Behavior Description
Hand Assistance System	Frame	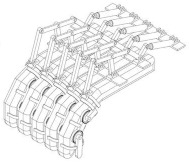	Fixed on the Back of the Hand	Used to Secure the Trainee’s Hand Back
	Hand Assistance Mechanism		The five finger assistive mechanisms correspond to each finger, with each mechanism including a hinged L-shaped link, a first link, and a second link.	Connected to the drive components, these mechanisms assist the fingers in pressing the piano keys.
Power Unit	Push Rod Motor	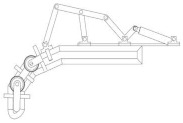	The push rods of the five pushrod motors are each hinged to the L-shaped linkages of the five finger assistance mechanisms, with the other end of the motors hinged to the top of the frame.	The motors push the five fingers of the user, passively pressing the correct piano keys to complete note playback.
Bracket Assembly	Expandable Component	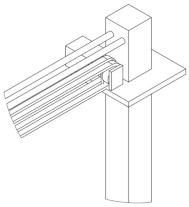	Includes the first and second telescopic tubes connected with expandable components.	Drives the learner’s wrist to move along the length of the piano to the corresponding key area based on the first instruction.
	Guide Rail		Fixedly connected to the opposing sides of the two telescopic components at both ends.	
Drive Component	Transverse Motor	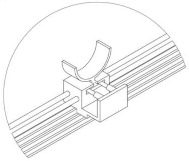	Fixed to the telescoping support.	Drives the movement of the user’s hand, positioning it in the correct range on the piano.
	Driven Wheel			Facilitates the left and right movement of the user’s wrists.

## Data Availability

No new data were created or analyzed in this study. Data sharing is not applicable to this article.
